# A pilot trial on lithium disilicate partial crowns using a novel prosthodontic functional index for teeth (FIT)

**DOI:** 10.1186/s12903-019-0957-4

**Published:** 2019-12-09

**Authors:** Edoardo Ferrari Cagidiaco, Simone Grandini, Cecilia Goracci, Tim Joda

**Affiliations:** 1 Department of Prosthodontics and Dental Materials, University of Siena, Siena, Italy; 2 Department of Periodontics, Universidad Complutense de Madrid, Madrid, Spain; 3 Department of Endodontics and Restorative Dentistry, University of Siena, Siena, Italy; 4 Department of Medical Biotechnologies, Policlinico Le Scotte, Viale Bracci 1, 53100 Siena, Italy; 5 Department of Reconstructive Dentistry, University Center for of Dental Medicine, Basel, Switzerland

**Keywords:** Partial crowns, Lithium disilicate, Randomized controlled trial

## Abstract

**Background:**

Lithium disilicate is now a well accepted material for indirect restorations. The aim of this trial was to evaluate two lithium disilicate systems using a novel prosthodontic Functional Index for Teeth (FIT).

**Methods:**

Partial adhesive crowns on natural abutment posterior teeth were made on sixty patients. Patients were divided into two groups: Group 1 IPS e.max press (Ivoclar-Vivadent, Schaan, Liecthestein), and Group 2 Initial LiSi press (GC Co., Tokyo, Japan). The restorations were followed-up for 3 years, and the FIT evaluation was performed at last recall. The FIT is composed of seven variables (Interproximal, Occlusion, Design, Mucosa, Bone, Biology and Margins), each of them are evaluated using a 0–1-2 scoring scheme, and is investigated by an oral radiograph and occlusal and buccal pictures. More in details, three variables have the three scores made on the presence or not of major, minor or no discrepancy (for ‘Interproximal’, ‘Occlusion’ and ‘Design’), presence or not of keratinized and attached gingiva (‘Mucosa’), presence of bone loss > 1.5 mm, < 1.5 mm or not detectable (‘Bone’), presence or not of Bleeding on Probing and or Plaque Index (‘Biology’), presence of detectable gap and marginal stain or not (‘Margins’). The Mann-Whitney ‘U’ test was used and the level of significance was set at *p* < 0.05. Also, “success” of the crowns (restoration in place without any biological or technical complication) and “survival” (restoration still in place with biological or technical complication) were evaluated.

**Results:**

Regarding FIT scores, all partial crowns showed a stable level of the alveolar crest without detectable signs of bone loss in the radiographic analysis. All other evaluated parameters showed a high score, between 1.73 and 2. No statistically significant difference emerged between the two groups in any of the assessed variables (*p* > 0.05). All FIT scores were compatible with the outcome of clinical success and no one restoration was replaced or repaired and the success rate was 100%.

**Conclusions:**

The results showed that it is possible to evaluate the clinical performance of partial crowns using FIT. The FIT proved to be an effective tool to monitor the performance of the restorations and their compatibility with periodontal tissues at the recall. The FIT can be really helpful for a standardized evaluation of the quality of the therapy in prosthodontic dentistry. The two lithium disilicate materials showed similar results after 3 years of clinical service.

**Trial registration:**

The study protocol was approved by the Ethical Committee of University of Siena (clinicaltrial.gov # NCT 01835821), ‘retrospectively registered’.

## Background

Due to the specific properties of lithium disilicate, particularly flexural strength, this restorative material is mainly indicated for single full and/or partial crowns [1–3]. Lithium disilicate provides high aesthetic results and, in comparison with porcelain and reinforced resin composites, its higher flexural strength makes it be preferable whenever the tooth defect exceeds a certain dimension [4, 5].

Lithium disilicate can be obtained using two different production processes: press technology and CAD/CAM technology. CAD/CAM technology is mainly used as chairside procedure, while the pressable technology is performed in the laboratory mainly using an analogic workflow. Pressed lithium disilicate results were very promising [6, 7] and recently the evaluation of a new lithium disilicate material (Initial LiSi press, GC) has been reported [8]. Only few clinical trials are available on lithium disilicate partial crowns, the majority of them being retrospective studies [9–11] and only one being a randomized controlled trial (RCT) [8].

Evaluation of clinical results of partial crowns on posterior teeth is usually performed following standardized parameters, such as Ryge and Snyder clinical parameters [12] or the modified FDI criteria [13]. The evaluation is usually performed after luting at baseline, and then at recalls after 1,6,12, 24, or 36 months. The modified FDI criteria evaluate several categories with some sub-categories [13]. Also, RCTs are done by blinded, calibrated and experienced dentists that can perform the follow-up evaluation [14, 15].

It must be pointed out that Ryge and Snyder clinical parameters and modified FDI criteria were initially defined for direct restorations, therefore there is the need to determine clinical criteria adequate to evaluate indirect restorations. Clinical criteria should reflect the patients’ perception of the restorations, fulfilling teaching purposes and being easily applicable in daily practice. In order to ease the process of drafting a proper treatment plan [16, 17], some classifications and prognosis evaluations have been proposed.

Recently, a novel Functional Implant Prosthodontic Score (FIPS) was proposed [18–21]; FIPS was based on 5 clinical variables evaluated crowns placed on implants with an oral radiograph and a buccal and an occlusal picture. Its potential to serve as an objective and reliable instrument in assessing implant success and restoration and periodontal outcome as perceived by patients, as well as identifying the possible risk of failure, comparing follow-up observations, providing an effective teaching tool was demonstrated. Similarly, FIT, that is a novel index for the assessment of the prosthetic results of lithium disilicate crowns, based on seven restorative-periodontal parameters, that evaluate crowns placed on natural abutments, and want to be a reliable and objective instrument in assessing single partial crown success and periodontal outcome as perceived by patients and dentists.

## Methods

The aim of this RCT was to evaluate the clinical performance of two lithium disilicate pressed systems using a novel Functional Index for Teeth (FIT), which is made up of seven clinical variables showing, among other things, the possible correlation with the level of appreciation perceived by the patients.

### Functional index for teeth (FIT)

A novel Functional Index for Teeth (FIT) was used (Table 2). Seven clinical variables have been collected and main prosthodontic and periodontal parameters were evaluated simultaneously (Interproximal Contacts and Papillae, Static and Dynamic Occlusion, Design Contour and Color, Quality and Quantity of Mucosa, Bone level in x-Ray, Biology related to Bleeding on Probing (BoP) and Plaque Index (PI) and Stain and Gap at Margins).

**Fig. 1:**
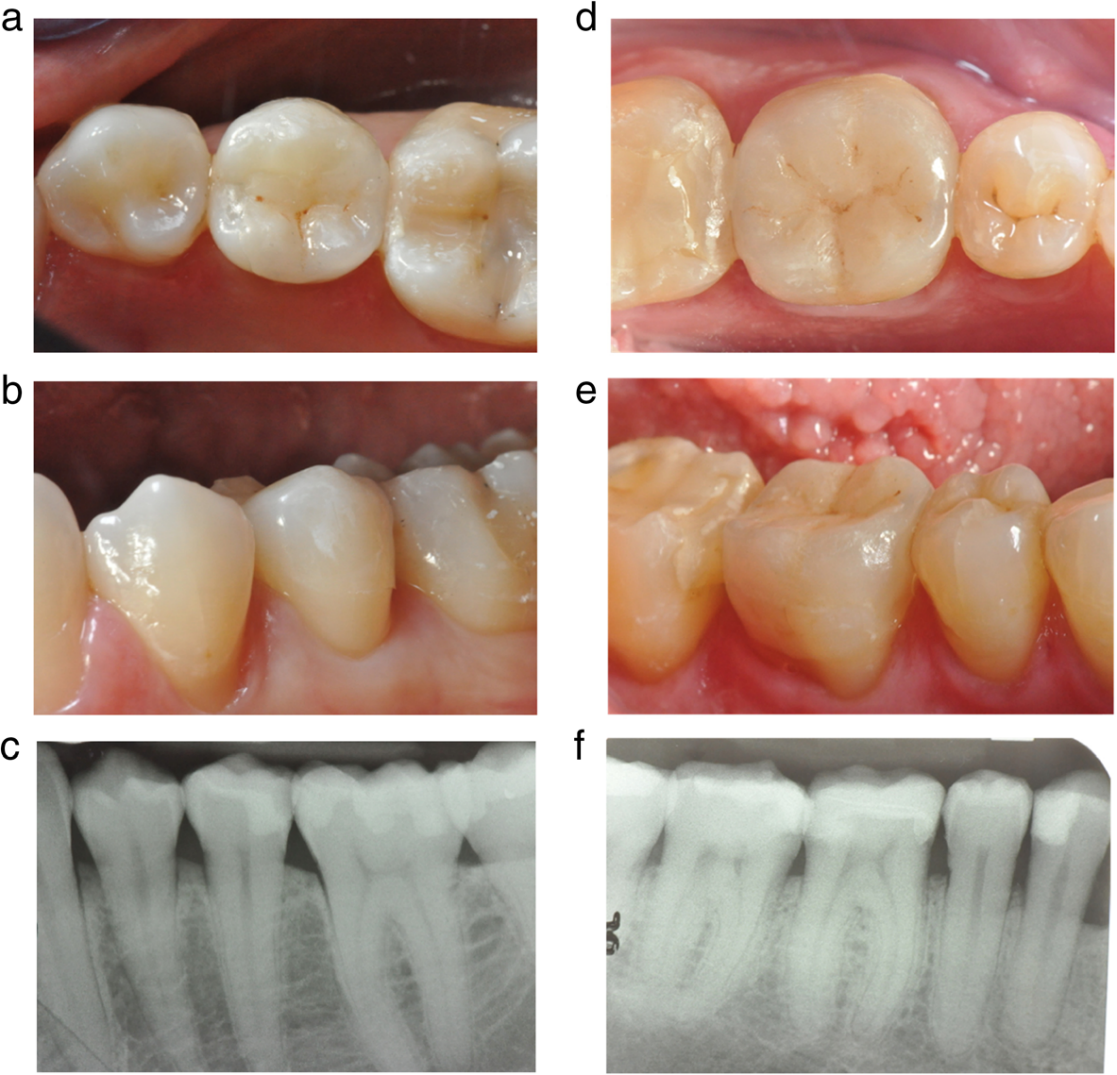
**a**, **b** and 1**c**. are related to a clinical case of Group 1 (the second premolar received a IPS e.max press restoration) while Fig. 1 **d**, **e** and **f** of Group 2 (the first molar received a GC Initial™ LiSi Press restoration). No technical or biological complications were observed at 3-year recall

The FIT evaluation was performed only at last recall (3-year follow-up) by an experienced operator (Fig. 1 a-f).

The null hypothesis tested in this clinical study was that there was no statistically significant difference in the clinical performance of the two lithium disilicate systems. A sample of 60 patients in need of a single partial crown on posterior teeth (upper and lower premolars and molars), accessing the Department of Prosthodontics and Dental Materials of the University of Siena, Italy, in the time period between September 2015 and January 2016 were included in the study. Selected patients, periodontally healthy or successfully treated in need for one posterior restoration, had a mean age of 37 (±7.5) years (between 18 and 70) (14F,16M). Exclusion criteria were: age < 18 years, pregnancy, disabilities, prosthodontic restoration of the tooth, spontaneous sensitivity, pulpitic, non-vital or endodontically treated teeth, (chronic) periodontitis, deep defects (close to pulp, < 1 mm distance) or pulp capping, heavy occlusal contacts or history of bruxism, systemic disease or severe medical complications, allergic history concerning methacrylates, rampant caries, xerostomia, lack of compliance, language barriers, plaque index higher than 20.

Patients written consent to the trial was obtained after having provided a complete explanation of the aim of the study. The study protocol was approved by the Ethical Committee of University of Siena (clinicaltrial.gov # NCT 01835821). All procedures performed in this study involving human participants were in accordance with the ethical standards of the Institutional and National Research Committee and with the 1964 Helsinki declaration and its later amendments or comparable ethical standards. This study adheres to CONSORT guidelines.

### Randomization selection of the patients and masking of examiners

After recruitment, oral hygiene instructions were given to the patients and prophylaxis was performed to establish optimal plaque control and gingival health.

The clinical assessment of periodontal parameters such as probing pocket depths (PPD) [22], bleeding on probing (BoP) [23], and full-mouth plaque index (PI) [22] was performed.

All restorative procedures were carried out under local anesthesia (Articaine with 1:100.000 epinephrine) by the same experienced operator. Intraoral radiographs were also taken before starting the treatment. In order to standardize the radiographic examination, X-ray individual tray was made for each sample tooth of each patient, to be sure to have the radiogram in the same position at each recall.

Each participating patient was randomly assigned to one of the two experimental groups (*n* = 30), that were defined based on the material to be used for the restorative treatment:

Group 1: IPS e.max press (Ivoclar-Vivadent, Schaan, Lichtenstein).

Group 2: Initial LiSi press (GC Co., Tokyo, Japan).

Main characteristics of the two Lithium Disilicate materials were reported in Table 1.

**Table 1:** Mechanical properties of IPS e.max press and GC Initial™ LiSi press materials.

Properties (as provided by manufacturers)	Units	IPS e.max Press	Initial LiSi Press
Manufacturer	–	Ivoclar Vivadent	GC
Components	–	lithium disilicate crystals (approx. 70%), Li2Si2O5, embedded in a glassy matrix	lithium disilicate micro-crystals equally dispersed in a glass matrix
Crystal system	–	lithium disilicate - crystals measure 3 to 6 μm in length.	lithium disilicate - crystals measure 1.5 μm × 0.5 μm
Flexural Strength	MPa	433*	454*
Biaxial Flexural Strength	MPa	> 500	> 500
Vickers hardness		(HV10) 5900 ± 100 Mpa	600 HV
Chemical solubility	mg/cm2	40 ± 10	5.4 μg/cm^2^
Liner thermal expansion CTE	× 10–6/K	Coefficient of thermal expansion (100–400 °C) 10.15 ± 0.4 10^− 6^ K^− 1^ Coefficient of thermal expansion (100–500 °C) 10.55 ± 0.35 10^− 6^ K^− 1^	Liner thermal expansion CTE (25–500 °C) 9,8 × 10^− 6^ K^− 1^
Glass transition temperature	°C	560	520
Density	g/cm^3^	2.5 ± 0.1	2,4

*Internal data, University of Siena.

Treatment assignment was noted in the registration and treatment assignment form that was kept by the study. Allocation concealment was performed by using opaque sealed, sequentially numbered envelopes. The statistician made the allocation sequence by means of a computer-generated random list and instructed a different subject to assign a sealed envelope containing the type of lithium disilicate material to be used. The opaque envelope has been opened before material selection and communicated to the operator. At the 3-year recall blinding of the examiner has been applied.

### Clinical procedure

For standardization purposes, all clinical procedures were performed by the same trained operator. Following anesthesia, rubber dam was placed, all carious lesions were excavated, and any restorative material was removed. Preparation was performed using conventional diamond burs in a high-speed hand piece, with no bevel on margins. The preparation design was dictated by the extent of decay, pre-existing restorations and the preparation guidelines defined by the manufacturer of the restorative materials. The Residual Dentin Thickness (RDT) was evaluated on a periapical radiograph, and teeth with RDT thinner than 0.5 mm were excluded. Cavities’ preparation provided at least 0.5–1 mm space at the margin and 1.0–1.5 mm of clearance occlusally. Margins were mainly into enamel and only interproximal boxes had cervical margin below the cementum-enamel junction for no more than 1 mm. At least one cusp was covered. Teeth were kept vital.

Hybridization of dentin with adhesive material was done using Adhese Bond Ivoclar-Vivadent, Schaan, Liechtenstein, in Group 1 and G-Premio Bond, GC Co., Tokyo, Japan in Group 2, and then a thin layer of flowable has been applied on top (Tetric Flow, Ivoclar-Vivadent in Group 1 and Genial Flow, GC Co, in Group 2). After the final preparation, an impression of the prepared tooth was taken with an elastomeric material (Exa’lence, GC Co.), and poured in stone (FujiRock, GC Co.). The restoration was then waxed and pressed in lithium disilicate, strictly following the manufacturer’s instructions. A temporary restoration of the prepared tooth was provided and after one week the lithium disilicate restoration was luted following manufacturer’s instructions. The intaglio surface of the restoration was etched with 10% hydrofluoric acid for 1 min, silanized with Monobond Plus (Ivoclar-Vivadent, Schaan, Lietchtenstein) in Group 1 and G-Multi Primer (GC Co.) in Group 2, and then luted using MultiLink Sprint (Ivoclar-Vivadent) in Group 1 and LinkForce (GC Co.) in Group 2. During cementation proper tooth isolation was provided by rubber dam.

### Follow-up

All patients were enrolled in a dental hygiene program in which recalls were planned every 6 months. A clinical exam and standardized intraoral radiographs were performed immediately after the seating of the crowns (baseline), as well as after 1, 2, and 3 years of clinical service (follow-up).

### Outcome variables

“Success” was set when the restoration was in place at last recall without any biological or technical complication, whilst “Survival” when the restoration was still in place at last recall but with biological or technical complications that needed to be treated and/or the crown to be remade. “Failure” was set when the restoration was not in place anymore at last recall or, because of mechanical or biological complications, needed to be replaced.

### Statistical analysis

The Mann-Whitney ‘U’ test was applied to verify the statistical significance of the difference between the two groups in the scores recorded for each assessed variable. The level of significance was set at *p* < 0 .05. The statistical analysis was handled by the PASW Statistics 18 software (IBM, Armonk, NY, USA).

## Results

The recall rate of patients was 100% and for that no loss to follow up was recorded. Survival and success rates were 100%. No technical or biological complications were observed during follow-up.

Clinical examinations of periodontal parameters showed mean scores for PI of 18.0 (SD 2.5; range: 16–21) at baseline and 17.5 (SD 1.0) (range: 16–20) at 1- year follow-up, PPD of 3.4 (SD 0.5 mm; range: 1–4) and 3.2 (SD 0.5 mm; range: 1–4), and a mean score for BoP of 18.4 (SD 2.2; range: 17–24) and 16.6 (SD 1.4; range: 16–22), respectively. At last recall, scores of periodontal parameters showed a proper maintenance of periodontal health thanks to professional recall program and home maintenance of patients.

At the 3-year follow up, the mean total FIT score was 13.26 and 13.66 for Group 1 and 2 (range: 10–14) respectively (Table 2). All partial crowns showed a stable level of alveolar crest without signs of bone loss at the radiographic analysis. Therefore, the variable radiographic “bone “level demonstrated the most consistent result and the highest scores, with a mean value of 2 (range: 2–2) in both groups. Similarly, the mean scores recorded for the variables “static and dynamic occlusion” and “quality and quantity of mucosa” were 2 (range: 2–2) in Group 1 and 1.9 in Group 2 (range: 1–2). In contrast, mean scores for “design contour and color” were 1.86 (SD 0.7) in Group 1 (range: 1–2), and 2 (range: 2–2) in Group 2; “mucosa “2 (range: 2–2) in Group 1 and 1,93 (SD 0.2; range 1-2) in Group 2; “interproximal contacts and papillae” 1.73 (SD 0.7; range: 1–2) in Group 1 and 2 (range: 2–2) in Group 2; “biology” scored 1.93 (SD 0.3; range 1–2) in both Groups; and “stain and gap at margins” was 1.73 (SD 0.8; range 0–2) in Group 1 and 1.86 (SD 0.7; range 1–2) in Group 2 were the most challenging to satisfy (Table 3).

**Table 2:** Functional Index for Teeth Prosthodontic (FIT)

Scoring Scheme	0	1	2
Interproximal	major discrepancy	minor discrepancy	no discrepancy
Contacts & Papillae	(2x incomplete)	(1x complete)	(2x complete)
Occlusion	major discrepancy	minor discrepancy	no discrepancy
Static & Dynamic	(supra-contact)	(infra-occlusion)	
Design	major discrepancy	minor discrepancy	no discrepancy
Contour & Color	(contour)	(color)	
Mucosa	non-keratinized	non-keratinized	keratinized
Quality & Quantity	non-attached	attached	attached
Bone	radiographic bone loss	radiographic bone loss	radiographic bone loss
X-Ray	> 1.5 mm	< 1.5 mm	not detectable
Biology	BoP and PI present	BoP present	no clinical impairment
BoP & PI			
Margins	detectable gap and visible stain	detectable gap or visible stain	no clinical impairment
Gap & Stain			
Max Score			**14**

**Table 3:** Radiographic and clinical scores based on FIT for each group

Variables	Group 1 IPS e.max (n = 30) (total) (median)	Group 2 GC Initial™ LiSi (n = 30) (total) (median)	Total Score Each Outcome
Interproximal Contacts & Papillae	26 (1.73)	30 (2)	**(56)**
Occlusion Static & Dynamic	30 (2)	29 (1.93)	**(59)**
Design Contour & Color	28 (1.86)	30 (2)	**(58)**
Mucosa Quality & Quantity	30 (2)	29 (1.93)	**(59)**
Bone X-Ray	30 (2)	30 (2)	**(60)**
Biology BoP -& PI	29 (1.93)	29 (1.93)	**(58)**
Margins Gap & Stain	26 (1.73)	28 (1.86)	**(54)**
Total Score Each Group	**199 (13.26)**	**205 (13.66)**	

No statistically significant difference emerged between the two groups in any of the assessed variables (*p* > 0.05).

## Discussion

Some clinical parameters such as Ryge and Snyder criteria [12] or the modified FDI criteria [13–15, 24] are commonly used as evaluation method of clinical trials. The Ryge and Snyder parameters evaluate post-operative sensitivity, retention, marginal gap, marginal discoloration, fracture, interproximal contacts and secondary caries, scoring each parameter in alpha, beta, charlie and delta and are the most used clinical criteria to evaluate direct restorations. The modified FDI criteria evaluate several categories such as aesthetic, functional and biological properties with four sub-categories each. Each sub-category is then divided into 5 quality scores from clinically excellent/very good to clinically poor, for a total of 16 criteria that might not be all used in the same case [13]. A calibration by e-calib system of the FDI criteria is available and its main goals were to efficiently train and calibrate clinical dental research workers using e-learning tools, to reduce the variability of the outcome of dental restorations in clinical studies using standardized assessment criteria, to better compare the results of clinical trials on dental restorations among different clinics in the world, to render clinical calibration programs more efficient, to improve daily clinical practice and to be used as a teaching tool in dental schools [15].

The FIT evaluation was proposed for the first time in the present study and is based on 7 clinical parameters: interproximal, occlusion, design, mucosa, bone, biology, margins. Although its targets resemble the ones of the modified FDI criteria, that is limited to the tooth and the restoration without evaluating the periodontal tissues, FIT can also evaluate the periodontal tissues behavior by ‘Interproximal’, ‘Mucosa’, ‘Bone’ and ‘Biology’ parameters and is a more user-friendly and straightforward method for the clinician to be applied in everyday practice.

The fact that RCTs are carried out by blinded, calibrated, and experienced dentists that perform the follow-up evaluations in specialized centers [24] might be considered as a limit. In fact, it is still under discussion in the dental scientific community whether thus conducted RCTs accurately represent the reality of daily practice. One of the main goals of FIT is to make practitioners more familiar with the core idea of RCTs by getting them into the habit of scoring their restorations, following the evolution of clinical parameters at each recall.

The two novel proposed classifications (FIT for single crowns on natural abutments and FIPS for single crowns on fixtures) evaluate individual teeth with special regard to their periodontal conditions in order to formulate an appropriate treatment plan [16, 17]. FIT, on the other hand, was conceived for single restorations and, consequently, it can be applied to any indirect restorations.

It must be considered that the operator’s experience can be a key factor when a Randomized Controlled Trial (RCT) is done and FIT is applied; However, in order to explain the high success rate found in this pilot RCT, the oral hygiene maintenance (professional and at home) of the selected patients in combination with the experience and skill of the operator must be considered.

The FIT scores recorded in this RCT were high for all parameters and no statistically significant differences were found between the two tested lithium disilicate materials. Such findings lead to acceptance of the formulated null hypothesis. The lack of differences between the two pressed lithium disilicate materials showed that the new system, which has been recently launched into the market (Initial LiSi Press), can clinically perform as well as e.max pressed system (IPS e.max press), that has instead been marketed for many years.

It must be pointed out that about IPS e.max press several clinical studies are available in the literature [25–29]. There is consensus that IPS e.max press (also with the previous name of Empress 2) has good enough longevity when used to restore single tooth after 5 years (survival of 90%) [25, 26] and 71% after 10 years of clinical service [27, 28]. Particularly relevant is the recently published report by Malament [29] in which was found out that pressed lithium disilicate restorations (Empress 2) survived successfully over the 10.4 period studied with an overall failure rate below 0.2% per year and primarily confined to molar teeth. It can be speculated that also in this study [29] skill and knowledge of the operator and oral hygiene regime can contribute to the impressive success rate.

Regarding Initial LiSi press, only one prospective clinical study is already available and showed 100% survival after 3 years [8]. Long term RCT results are need in order to evaluate longevity under clinical function of Initial LiSi press.

The limited number of restorations for each group and the relatively short time of observation might be considered as a shortcoming of this study, possibly affecting the power of the statistical tests. Also, it must be point out that, accordingly with exclusion criteria, a category of patients without any health issue were really selected. This might be considered a partial limitation of this study.

Usually RCTs are being conducted on larger samples, and they might compare Ryge and Snyder clinical parameters with the modified FDI and FIT scores. Another possible limitation of the present RCT is the reduced number of tested materials; a similar RCT comparing several restorative materials (e.g reinforced resins in different formulations) in a wider number of patients is ongoing.

## Conclusions

The findings of this study showed that FIT score can be a reliable tool to rate the clinical outcome of posterior partial crowns over time. FIT score can also be useful to monitor any possible early failure and to standardize follow-up recalls. Furthermore, the two lithium disilicate materials tested in this RCT showed comparable clinical performances, with high success rate after 3-year of service.

## Data Availability

The datasets used and/or analysed during the current study are available from the corresponding author on reasonable request after approval of all other authors.

## Abbreviations

BoP: bleeding on probing
FDI: Federation Dental International
FIPS: Functional Implant Prosthodontic Score
FIT: Functional Index for Teeth
PI: full-mouth plaque index
PPD: probing pocket depths
RCT: Randomized Controlled Trial
RTD: Residual Dentin Thickness

## References

[1] Monaco C, Caldari M, Scotti R (2013). AIOP clinical research group: Clinical evaluation of 1,132 zirconia-based single crowns: a retrospective cohort study from the AIOP clinical research group. Int J Prosthodont.

[2] Vichi A, Sedda M, Del Siena F, Louca C, Ferrari M (2013). Flexural resistance of Cerec CAD/CAM system ceramic blocks. Part 1: Chairside materials. Am J Dent.

[3] Sedda M, Vichi A, Del Siena F, Louca C, Ferrari M (2014). Flexural resistance of Cerec CAD/CAM system ceramic blocks. Part 2: Outsourcing materials. Am J Dent.

[4] Manhart J, Chen H, Hamm G (2004). Review of the clinical survival of direct and indirect restorations in posterior teeth of the permanent dentition. Oper Dent.

[5] Culp L, McLaren EA (2010). Lithium disilicate: the restorative material of multiple options. Compend Contin Educ Dent.

[6] Ritter RG (2010). Multifunctional uses of a novel ceramic-lithium disilicate. J Esthet Rest Dent.

[7] Yu J, Gao J, Guo J, Li L, Zhao Y, Zhang S (2016). Clinical outcomes of different types of tooth-supported bilayer lithium disilicate all-ceramic restorations after functioning up to 5 years: a retrospective study. J Dent.

[8] Ferrari M, Ferrari Cagidiaco E, Goracci C, Sorrentino R, Zarone F, Grandini S, Joda T (2019). Posterior partial crowns with or without posts: a 3-year follow up. J Dent.

[9] Cortellini D, Canale A (2015). Characteristics of Lithium Disilicate Crowns Bonded on Abutments with Knife-Edge and Large Chamfer Finish Lines after Cyclic Loading. J Prosthodont.

[10] Schmitz JH, Beani M (2016). Effect of different cement types on monolithic lithium disilicate complete crowns with feather-edge preparation design in the posterior region. J Prosthet Dent.

[11] Schmitz JH, Cortellini D, Granata S, Valenti M (2017). Monolithic lithium disilicate complete single crowns with feather-edge preparation design in the posterior region: A multicentric retrospective study up to 12 years. Quintessence Int.

[12] Ryge G, Snyder M (1973). Evaluating the clinical quality of restorations. J Am Dental Assoc.

[13] Hickel R, Peschke A, Tyas M, Mjor IA, Baybe S, Peters M, Hiller KA, Randall R, Vanherle G, Heintze SD (2010). FDI world dental federation: clinical criteria for the evaluation of direct and indirect restorations-update and clinical examples. Clin Oral Investig.

[14] Hickel R, Roulet JF, Bayne S, Heintze SD, Mjor IA, Peters M, Roussen V, Randall R, Schmalz G, Tyas M, Vanherle G (2007). Recommendations for conducting controlled clinical studies of dental restorative materials. Science committee project 2/98--FDI world dental federation study design (part I) and criteria for evaluation (part II) of direct and indirect restorations including onlays and partial crowns. J Adhes Dent.

[15] Hickel R, Peschke A, Tyas M, Mjor IA, Baybe S, Peters M, Hiller KA, Randall R, Vanherle G, Heintze SD (2010). FDI world dental federation - clinical criteria for the evaluation of direct and indirect restorations. Update and clinical examples. J Adhes Dent.

[16] Kwok V, Caton J (2007). Prognosis revisited: a system for assigning periodontal prognosis. J Periodontol.

[17] Samet N, Jotkowitz A (2009). Classification and prognosis evaluation of individual teeth-a comprehensive approach. Quintessence Int.

[18] Joda T, Ferrari M, Bragger U (2017). A prospective clinical cohort study analyzing single-unit implant crowns after 3 years of loading: introduction of a novel functional implant Prosthodontic score (FIPS). Clin Oral Implants Res.

[19] Joda J, Zarone F, Zitzmann NU, Ferrari M (2018). The functional implant Prosthodontic score (FIPS): assessment of reproducibility and observer variability. Clin Oral Investig Clin Oral Investig.

[20] Joda T, Ferrari M, Bragger U (2017). Monolithic implant-supported lithium disilicate (LS2) crowns in a complete digital workflow: a prospective clinical trial with a 2-year follow-up. Clin Impl Dent Relat Res.

[21] Joda T, Bragger U, Zitzmann NU (2019). CAD/CAM implant crowns in a digital worflow: five-year follow-up of a prospective clinical trial. Clin Implant Dent Relat Res.

[22] Löe L, Silness J (1963). Periodontal disease in pregnancy. I prevalence and severity. Acta Odontol Scand.

[23] Ainamo J, Bay I (1975). Problems and proposals for recording gingivitis and plaque. Int Dent J.

[24] Hickel R, Roulet JF, Bayne S, Heintze SD, Mjor IA, Peters M, Roussen V, Randall R, Schmalz G, Tyas M, Vanherle G (2007). Recommendations for conducting controlled clinical studies of dental restorative materials. Clin Oral Investig.

[25] Solá-Ruiz MF, Lagos-Flores E, Román-Rodriguez JL, Highsmith Jdel R, Fons-Font A, Granell-Ruiz M (2013). Survival rates of a lithium disilicate-based core ceramic for three-unit esthetic fixed partial dentures: a 10-year prospective study. Int J Prosthodont.

[26] Layton DM, Clarke M (2013). A systematic review and meta-analysis of the survival of non-feldspathic porcelain veneers over 5 and 10 years. Int J Prosthodont.

[27] Teichmann M, Göckler F, Weber V, Yildirim M, Wolfart S, Edelhoff D (2017). Ten-year survival and complication rates of lithium-disilicate (empress 2) tooth-supported crowns, implant-supported crowns, and fixed dental prostheses. J Dent.

[28] Teichmann M, Göckler F, Rückbeil M, Weber V, Edelhoff D, Wolfart S (2019). Periodontal outcome and additional clinical quality criteria of lithium-disilicate restorations (empress 2) after 14 years. Clin Oral Investig.

[29] Malament KA, Natto ZS, Thompson V, Rekow D, Eckert S, Weber HP (2019). Ten-year survival of pressed, acid-etched e.max lithium disilicate monolithic and bilayered complete-coverage restorations: performance and outcomes as a function of tooth position and age. J Prosthet Dent.

